# Activated Signaling Pathways and Targeted Therapies in Desmoid-Type Fibromatosis: A Literature Review

**DOI:** 10.3389/fonc.2019.00397

**Published:** 2019-05-17

**Authors:** Milea J. M. Timbergen, Ron Smits, Dirk J. Grünhagen, Cornelis Verhoef, Stefan Sleijfer, Erik A. C. Wiemer

**Affiliations:** ^1^Department of Surgical Oncology, Erasmus MC-University Medical Center, Rotterdam, Netherlands; ^2^Department of Medical Oncology, Erasmus MC-University Medical Center, Rotterdam, Netherlands; ^3^Department of Gastroenterology and Hepatology, Erasmus MC-University Medical Center, Rotterdam, Netherlands

**Keywords:** desmoid-type fibromatosis, signaling pathways, desmoid tumor, *CTNNB1* mutation, review, ß-catenin, targeted therapies

## Abstract

Desmoid-type fibromatosis (DTF) is a rare, soft tissue tumor of mesenchymal origin which is characterized by local infiltrative growth behavior. Besides “wait and see,” surgery and radiotherapy, several systemic treatments are available for symptomatic patients. Recently, targeted therapies are being explored in DTF. Unfortunately, effective treatment is still hampered by the limited knowledge of the molecular mechanisms that prompt DTF tumorigenesis. Many studies focus on Wnt/β-catenin signaling, since the vast majority of DTF tumors harbor a mutation in the *CTNNB1* gene or the *APC* gene. The established role of the Wnt/β-catenin pathway in DTF forms an attractive therapeutic target, however, drugs targeting this pathway are still in an experimental stage and not yet available in the clinic. Only few studies address other signaling pathways which can drive uncontrolled growth in DTF such as: JAK/STAT, Notch, PI3 kinase/AKT, mTOR, Hedgehog, and the estrogen growth regulatory pathways. Evidence for involvement of these pathways in DTF tumorigenesis is limited and predominantly based on the expression levels of key pathway genes, or on observed clinical responses after targeted treatment. No clear driver role for these pathways in DTF has been identified, and a rationale for clinical studies is often lacking. In this review, we highlight common signaling pathways active in DTF and provide an up-to-date overview of their therapeutic potential.

## Introduction

Desmoid-type fibromatosis (DTF) is a clonal fibroblastic proliferation of the soft tissues that arises in musculoaponeurotic structures (1). It has a mesenchymal origin since DTF tumors express cell surface markers and genes that are characteristic of mesenchymal stem cells (2). The incidence in the Dutch population is 5 patients per million people per year (3). Unfortunately worldwide epidemiological data is lacking. The abdominal wall and the trunk are the most common localizations and symptoms can vary, depending on tumor location and size (4, 5). Roughly two types can be distinguished; sporadic and hereditary DTF. The first type is considered to be a monoclonal disorder, since it derives from a single progenitor cell (6). This “sporadic” type is commonly localized extra-abdominally or in the abdominal wall (5). The precise etiology of sporadic DTF remains tenuous. Several studies report correlations with (spontaneous or iatrogenic) trauma and hormonal status (7–10). The hereditary type occurs more frequent in patients with familial adenomatous polyposis (FAP), and causes intra-abdominal DTF tumors. This DTF type is an autosomal dominant disorder caused by germline mutation of the *adenomatous polyposis coli* (*APC*) gene, and is associated with the formation of hundreds of colon polyps which can transform into malignant colorectal tumors in time [reviewed by De Marchis et al. (11) and Lips et al. (12)]. The cumulative rate of DTF in FAP patients is 20.6% at 60 years of age (13).

Desmoid-type fibromatosis is considered to be a borderline tumor because of its incapability to metastasize (1). The mortality of this disease is low and seldom described in literature. However, local aggressive growth can cause significant morbidity by infiltrating surrounding structures, causing pain or functional loss. Currently, “wait and see” is the first line therapy in case of asymptomatic DTF. Several retrospective studies report that a minority of patients on a “wait and see” protocol experience progression and that progression usually occurs within 2 years after tumor development (14). Additionally, up to one third of patients experience disease regression without any form of treatment (15–17). Three prospective studies investigating a “wait and see” approach (NCT02547831, Italy; NTR 4714, the Netherlands; NCT01801176, France) examine the natural growth behavior of DTF and their relationship with *CTNNB1* mutations (18–20). Surgery is the treatment of choice in case of failure of the “wait and see” management (21). Radiotherapy is mainly used as an adjuvant treatment in case of incomplete surgical resection. Radiotherapy, as a single treatment modality, may be considered for patients in whom local control is the primary treatment goal (21). When both surgery and radiotherapy are not an option due to tumor localization (e.g., near vital structures), or because of comorbidities, several other treatment options are available like local cryoablation and partial systemic chemotherapy via isolated limb perfusion (21). Although not widely used, as the evidence for their effect in DTF is only based on small patient series, some patients benefit from these local therapies for example when limb salvage is the treatment goal (22–25). Besides targeted drugs, other systemic options include more classic chemotherapeutic compounds like vinblastine, vinorelbine, methotrexate, doxorubicin, dacarbazine, either as a single agent or as combination therapy (21). Although most studies describe small retrospective case series and include patients who received other treatments prior to their cytotoxic treatment, multiple studies indicate a potential effect of these drug regimens (26–29).

The aggressive growth behavior, in combination with the high recurrence rate, creates the need for effective drugs targeting the molecular mechanisms that drive tumorigenesis (30, 31). This is especially true for large, symptomatic tumors which cannot be treated surgically or with radiotherapy. As stated above, several systemic options are available with variable efficacy in different patients, but no consensus about the nature and the sequence of systemic treatments has been established (21). As of yet, the exact working mechanisms of these systemic agents in DTF remain unclear.

A better understanding of the molecular mechanisms that prompt tumorigenesis and influence DTF progression will contribute to the development and implementation of new targeted therapies. This review comprehensively screened the available literature regarding active cell signaling and biochemical pathways and reviews pathway-specific targeted drugs investigated in DTF. Additionally, the challenges of DTF research, as well as the future perspectives, are discussed. The abbreviations used in the text, tables and figures are explained in Appendix 1.

## The Wnt/β-Catenin Signaling Pathway in Desmoid-Type Fibromatosis

### The Wnt/β-Catenin Signaling Pathway

The canonical Wnt/β-catenin pathway coordinates cell fate decisions during the developmental process and in adult homeostasis. Target genes of this signaling pathway are involved in regulating the balance between self-renewal, differentiation, apoptosis, and in stem cell maintenance [reviewed by Nusse and Clevers (32) and Steinhart and Angers (33)]. Activation of the Wnt/β-catenin pathway involves a Wnt ligand binding to the transmembrane receptor Frizzled, forming a complex with a co-receptor that is the LDL receptor-related protein 5 or 6 (LRP5 and LRP6). The β-catenin protein is a key mediator in the Wnt/β-catenin signaling pathway, and its stability is normally regulated by a degradation complex consisting of the tumor suppressor APC, a scaffolding protein axin, and two constitutively active serine-threonine kinases i.e., casein kinase 1α (CK1α/δ), and glycogen synthase kinase 3 (GSK3). Within this complex, β-catenin is sequentially phosphorylated by CK1 and GSK3 on serine/threonine residues (Ser45, Thr41, Ser37, Ser33), thus forming a docking site for the E3 ubiquitin ligase; β-TrCP. This ubiquitinylates β-catenin which is subsequently degraded by the proteasome. Activation of the Wnt/β-catenin pathway by binding of the Wnt ligand to the frizzled/LRP heterodimer recruits the degradation complex to the membrane via the disheveled protein (DVL) disrupting the degradation complex and consequently the phosphorylation of β-catenin, leading to its stabilization and translocation into the nucleus. In the nucleus it operates as a transcriptional activator, bound to members of the T-cell factor/lymphoid enhancer factor (TCF/LEF) transcription factor family, and possibly to other co-activators of Wnt target genes [reviewed by Nusse and Clevers (32)].

### The Wnt/β-Catenin Signaling Pathway in Cancer

The Wnt/β-catenin signaling pathway contributes to cancer by promoting progression of cells through the cell cycle, by inhibiting of apoptosis via the expression of anti-apoptotic genes, by affecting cell proliferation via the expression of growth factors and their corresponding receptors, by influencing cell motility through the expression of cell adhesion and extracellular matrix proteins and via stem cell maintenance [reviewed by Nusse and Clevers (32)]. Aberrant signaling of the Wnt/β-catenin pathway has been implicated in several epithelial tumors [e.g., colorectal carcinoma (34) and endometrial carcinoma (35)] and in mesenchymal tumors [e.g., osteosarcomas (36, 37), malignant fibrous histiocytomas and liposarcomas (38)].

### The Wnt/β-Catenin Signaling Pathway in Desmoid-Type Fibromatosis

The relationship between the Wnt/β-catenin signaling pathway and DTF has been extensively studied. It is believed that this pathway is crucial to DTF pathogenesis because of the fact that the vast majority (about 85%) of DTF tumors harbor a mutation in exon 3 of the *CTNNB1* (β-catenin) gene, making the protein more resistant to proteolytic degradation (39–41). Less frequently, loss-of-function mutations in the *APC* tumor suppressor gene are observed, most commonly in the context of FAP (12). In both cases, β-catenin translocates into the nucleus aberrantly activating target genes. This nuclear accumulation can be determined by immunohistochemistry (IHC), and serves as a diagnostic tool differentiating DTF from other bone-, soft tissue and fibrous tumors (42). The group of wild-type (WT) ß-catenin DTF, comprises about 15% of all DTF tumors, and is defined as “having no *CTNNB1* mutations in exon 3.” The number of DTF patients assigned to this group decreases over time since next generation sequencing is able to detect β-catenin mutations located on exon 3, in tumors where the traditional Sanger sequencing method is not sensitive enough (43, 44).

Interestingly, the β-catenin mutations observed in DTF are almost exclusively confined to residues T41 and S45, while alterations at other N-terminal phosphorylation residues, that is D32-S37, are rarely observed. Recently, Rebouissou et al. showed in liver cancers that the T41 and S45 mutants activate the pathway only weakly compared to others (45). Apparently, this weak activation is ideal for DTF outgrowth in line with the “just-right” signaling hypothesis that postulates that each tumor type selects for an optimal level of β-catenin signaling that is ideal for tumor initiation and progression (46). In accordance, the APC mutant proteins observed in DTF retain some functionality in regulating β-catenin levels. The specific β-catenin mutation may be of clinical relevance since several groups reported a higher recurrence rate in CTNNB1 S45F mutated DTF tumors compared to other CTNNB1 (T41A) mutated tumors and WT DTF (30, 47–49). This issue is however still under debate as others have reported contradictory results (41, 50).

Using a β-catenin reporter assay in primary DTF cultures, Tejpar et al. validated the enhanced β-catenin signaling present in DTF. They also showed that in the nucleus, β-catenin is mainly associated with TCF7L1 (also known as TCF3) to regulate target genes. Expression of TCF7 (TCF1) and LEF1 could not be identified, while solely a minority of DTF samples expressed TCF7L2 (TCF4) (51). Others found that several matrix metalloproteinases (MMP-3, MMP-7, and MMP-9) are expressed in DTF implying a role for MMP's in DTF invasiveness (52, 53). In fact Kong et al. showed that MMP inhibition decreases tumor invasion and motility (52). Matono et al. showed that MMP7 is more abundantly expressed in *CTNNB1* mutated DTF compared to *CTNNB1* WT, and hypothesized a correlation between MMP7 and prognosis, as previously was demonstrated in pancreatic cancer (54, 55). The MMP-inhibitor ilomastat (galardin/GM6001) was investigated in two studies, showing a decrease in DTF-cell (human and murine) migration and invasion capability (52, 53). In *Apc*^+/^*Apc*^1638N^ mutant mice, DTF tumor volume was decreased (Table 1) (52).

**Table 1:** Overview of drugs used in *in vitro*/*vivo* studies targeting a signaling pathway in DTF.

**Drug**	**References**	**Setting**	**Effect**
**WNT/ß-CATENIN SIGNALING PATHWAY**			
*MMP inhibitor* Ilomastat/Galardin (GM6001)	(52)	*Apc*^+^*/Apc^1638*N*^* mice Murine PCC from DTF and NF	↓ DTF cell invasion and motility ↓ Tumor volume in mice
	(53)	Human PCC from DTF and normal fascia	↔Cell growth ↓ DTF cell invasion
*NSAID* Sulindac/Indomethacin/DFU	(56)	Human PCC from DTF and normal marginal tissues *Apc^+^/Apc*^1638N^-Cox2*^−/−^* and *Apc^+^/Apc*^1638N^ *-*Cox2*^+/+^* mice	↓ DTF cell and NF proliferation ↔ Apoptosis in DTF cells and NF ↔ Tumor number in mice (sulindac) ↓ Tumor volume in mice (sulindac)
*NSAID* Sulindac	(57)	Human PCC from DTF, CRC	↓ DTF cell growth ↔ Cell morphology
*NSAID* Piroxicam (+DFMO)	(58)	*Apc^−/+^p53^+/−^*, *Apc^+/+^p53^+/−^*, *Apc^−/+^p53^+/+^* mice	↓ DTF tumor number
*Angiostatic factor* Endostatin	(59)	Human PCC from FAP-related DTF and CRC	↑ Apoptosis (CRC cultures) ↑ Cell death (DTF cultures)
*Benzoxazocine* Nefopam	(60)	Human PCC from DTF and NF *Apc^1638*N*^* mice	↓ Cell proliferation, modest change in apoptosis ↓ ß-catenin protein level ↓ tumor number and volume (mice)
**HEDGEHOG SIGNALING PATHWAY**			
*Hedgehog inhibitor* Triparanol	(61)	Human PCC from DTF *Apc^+/1638*N*^*; Gli2^+/−^ and *Apc^+/1638*N*^*; Gli2^+/+^ mice	↓ Tumor volume (*Apc^+/1638*N*^* mice) ↓ Number of tumors (*Apc^+/1638*N*^*; *Gli2^+/−^)* ↓ Number of tumor cells, viability, proliferation rate (DTF cells) ↔ Apoptosis (DTF cells)
**NOTCH SIGNALING PATHWAY**			
*γ-secretase inhibitor* PF-03084014	(62)	Human PCC from DTF	↓ Notch signaling (↓ NICD and Hes1 expression) ↑ Cell cycle arrest ↓ Cell growth, migration and invasion
**JAK/STAT SIGNALING PATHWAY**			
*Cytokines* Interferon-ß	(63)	Human PCC from DTF and NF *Apc/Apc^1638*N*^*, A*pc^1638*N*^;* *Ifnar1*^−/−^ and *Apc/Apc^1638*N*^; Ifnar1^+/+^*	↔ Apoptosis (human vs. murine DTF and NF) ↓ Cell proliferation (human/murine DTF and NF)
**PI3K/AKT/mTOR SIGNALING PATHWAYS**			
*Tyrosine kinase inhibitor* Sorafenib (±Everolimus)	(64)	Human PCC from DTF	↓ DTF cell proliferation and invasion (sorafenib) ↓ mTOR signaling [↓ phospho-S6K levels (everolimus)]
**GROWTH REGULATORY SIGNALING PATHWAYS**			
*Cytokines* TGF-ß1	(65)	Human PCC from DTF, fibroma, NF	↔ Cell proliferation in DTF, fibroma and NF cell culture ↑ GAG accumulation in extra-cellular matrix ↑ Collagen synthesis
	(66)	Human PCC from DTF and NF	↑ Active unphosphorylated fraction of ß-catenin
*Cytokines* rhEGF/rhTGF-α	(67)	Human PCC from DTF	Up- and down regulation of genes in response to stimulation with rhEGF/rhTGF-α
*Cytokines* rhEGF/AG1478/SB431542	(68)	Human PCC from DTF	↑ DTF cell motility (rhEGF)
**ESTROGEN DRIVEN PATHWAY**			
*Anti-estrogen* Tamoxifen/Toremifene	(69)	Human PCC from DTF	↓ Cell growth (tamoxifen ± estrogen) ↔ Cell growth (toremifene ± estrogen)
*Anti-estrogen* Toremifene	(70)	Human PCC from DTF, fibroma and NF	↔ Cell proliferation (^3^H-tymidine incorporation) ↓ GAG (DTF, fibroma and NF cultures) ↓ Collagen production (^3^H-proline incorporation) ↓ TGF-ß1 levels in culture medium ↓ TGF-ß1 mRNA expression levels ↓ TGF-ß1 receptor affinity
	(71)	Human PCC from DTF and NF	↑Cell death (DTF and NF culture) ↓ Collagen production (^3^H-proline incorporation) ↓ Procollagen α_1_ mRNA expression (DTF culture) ↓ Type I and III collagen ↑ Collagenase activity ↔ MMP-1, ↑ MMP-2, ↓ TIMP-1
	(72)	Human PCC from DTF, Gardner-syndrome related fibroblast and NF	↓ GAG synthesis and secretion ↓ Active TGF-ß1, ↔ total (active + latent) TGF-ß1 ↓ Number TGF-ß1 receptors (DTF cells) ↓ TNF-α production

*↓, decrease; ↑, increase; ↔, no effect; CRC, colorectal cancer; DFMO, Difluoromethylornithine; DFU, selective COX-2 blocker (5,5-dimethyl-3-(3-)4-(4-methylsulphonyl)phenyl-2(5H)-furon one); DTF, desmoid-type fibromatosis; FAP, familial adenomatous polyposis;, GAG, glycosaminoglycan; PCC, primary cell culture; NF, normal fibroblast; NSAID, non-steroidal anti-inflammatory drug*.

### Pharmacological Options Targeting the Wnt/β-Catenin Signaling Pathway

Although many studies implicated aberrant Wnt/β-catenin signaling in DTF tumorigenesis, therapeutic targeting of this pathway remains challenging. Wnt/β-catenin target-genes that do form attractive therapeutic targets in DTF are *cyclooxygenase* (*COX*), a member of the COX enzyme family (COX1 and COX2) and the *vascular endothelial growth factor* (*VEGF*), a protein that regulates angiogenesis. A role of COX in DTF has been indicated by the expression of COX2 and by the expression of phosphorylated, and thus activated, associated growth factors receptors, such as the platelet derived growth factor receptor α and ß (PDGF-α and PDGF-ß) (56, 73). Activation of their receptors (PDGFR-α/PDGFR-ß) takes place by an autocrine/paracrine loop and is initiated by COX2 overexpression due to Wnt/β-catenin deregulation (56, 73). Inhibition of COX with sulindac decreased cell proliferation in DTF cell culture and therefore forms an attractive therapeutic target in DTF, especially because COX inhibitors are already widely used in the clinic (56, 57). Halberg et al. reported decreased DTF tumor numbers in *Apc*^*Min*/+^*p53*^−/−^ mice treated with piroxicam, a drug which is a non-steroidal anti-inflammatory drug (NSAID) which works by inhibiting both prostaglandins and the COX enzyme (Table 1) (58).

**Figure 1:**
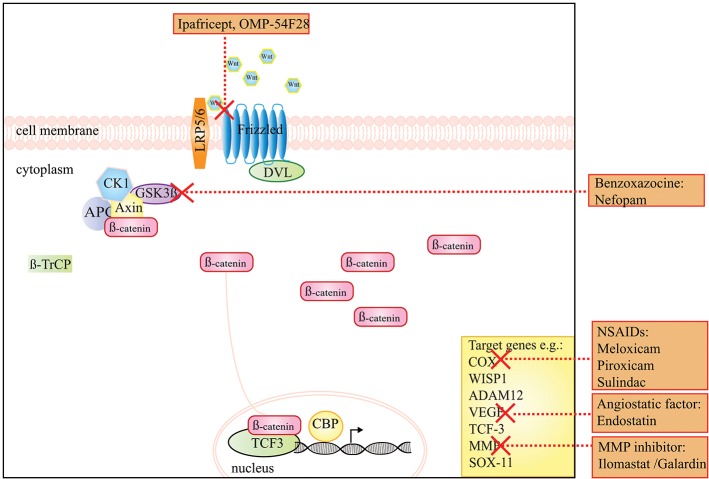
A schematic presentation of the Wnt/β-catenin signaling pathway and the drugs that target this pathway in DTF. The graph shows that ipafricept (OMP-54F28), inhibits Wnt signaling by acting as a decoy receptor inhibiting Wnt signaling through the Frizzled 9 receptor. NSAIDs, like meloxicam, the angiogenesis inhibitor endostatin and MMP inhibitors act on target genes of the Wnt signaling pathway. The drug Nefopam, a non-opioid analgesic drug of the benzoxazocine class suppresses the effect of high levels of β-catenin.

A preclinical study by Poon et al. used the non-opioid analgesic drug nefopam (benzoxazocine class), and reported a decrease in ß-catenin levels and cellular proliferation rate, as well as a reduction in tumor number and volume in *Apc*^+^^/^*Apc*^1638N^ mice (Table 1) (60). The working mechanism of this drug in DTF has not been entirely clarified yet, but is presumably due to an inhibition of serine-9-phosphorylation of GSK3-β (Figure 1) (60).

Overexpression of VEGF has been correlated with ß-catenin nuclear staining in DTF (74). Additionally, microvessel density, a phenomenon correlated to angiogenesis, was shown to be higher in samples with VEGF overexpression. This high vascularity potentially increases the growth potential of DTF tumors (74). These findings reveal a possible new treatment strategy for DTF by interfering with angiogenesis. Endostatin, an anti-angiogenic protein with the ability to inhibit the Wnt/β-catenin signaling pathway in colorectal cancer cells, directed the induction of cell death in primary FAP-associated DTF-cells in culture (59). Endostatin has been proven to be well-tolerated in a Phase 1 study, with minimal toxicities in patients with solid tumors other than DTF; however no studies report the use of endostatin for DTF in the clinic (75).

The blockage of Wnt/β-catenin signaling with the truncated Frizzled 9 receptor fused to the IgG1 Fc region (ipafricept, OMP-54F28), was recently tested in a Phase 1 study for solid tumors (Table 2). In this study, two patients with DTF were included that both exhibited stable disease, although it is unclear if this can be directly attributed to the treatment (76).

**Table 2:** Overview of drugs used in clinical trials targeting signaling pathways in DTF.

**Drug**	**References**	**Setting**	**Tumor type**	**N of DTF patients**	**Efficacy in DTF**
**WNT/ß-CATENIN SIGNALING PATHWAY**					
*Frizzled 9 receptor blocker* Ipafricept (OMP-54F28)	(76)	Phase 1	Advanced solid tumors (*n* = 26)	2	*N* = 2 *SD* (>6 months)
**NOTCH SIGNALING PATHWAY**					
PF-03084014	(77)	Phase 1	DTF	7	ORR 71.4% (95%CI 29–96.3%) *N* = 5 PR, median TTR 9.9 months *N* = 1 PD
	(78)	Phase 2	DTF	17	*n* = 5 PR (after a median of 32 cycles, 95 weeks), *n* = 11 *SD*
**PI3K/AKT/mTOR SIGNALING PATHWAYS**					
*Receptor Tyrosine Kinase inhibitor* Imatinib	(79)	Phase 2	DTF	40	*n* = 2 TS (<1 year) At 3 months: *n* = 1 CR, *n* = 3 PR, *n* = 28 *SD*, *n* = 5 PD 3-months NPRR 91% (95% CI 77–96), 6-months NPRR 80%, 12-months NPRR 67%
	(80)	Phase 2	DTF	51	At 2-months: *n* = 48 *SD* 2-months PFS 94%, 4-months PFS 88%, 1 year PFS 66%
	(81)	Phase 2	DTF	19	*n* = 3 PR, *n* = 4 *SD* (>1 year), 1-year disease control rate 36.8%, TTF 325 days
	(82)	Phase 2	Imatinib-sensitive tumors (*n* = 186)	20	DTF patients: *n* = 2 PR, *n* = 8 *SD, n* = 7 PD, *n* = 3 unknown. Median TTP 9.1 months (95% CI 2.9–17.0 months).
Imatinib (+nilotinib)	(83)	Phase 2	DTF	39	OS 100%, PAR at 6 months: 65% At 21 months: *n* = 7 PR, ORS 19% *n* = 8 imatinib + nilotinib due to PD under imatinib 3-months PAR 88%
Imatinib + gemcitabine (I + G) or Imatinib+ doxorubicin (I + D)	(84)	Phase 1	solid tumors (*n* = 16)	1 (I + G)	DTF patients: ceased treatment due to dose-limiting toxicity (grade 2 neutropenia)
Sunitinib	(85)	Phase 2	Non-GIST sarcomas (*n* = 53)	1	DTF patients: *n* = 1 NR
	(86)	Phase 2	advanced DTF	19	Median FU 20.3 months (1.8–50.7 months), 2-year PFS 74.7%, OS 94.4%. *n* = 5 PR, *n* = 8 *SD, n* = 3 PD, *n* = 3 not evaluable. Median duration of the response 8.2 months (range 2.0–17.3 months)
Sorafenib (+topotecan)	(87)	Phase 1	Pediatric solid malignancies (*n* = 13)	2	DTF patients: *n* = 1 PR
	(88)	Phase 3	Advanced and refractory DTF	87	2-year PFS of sorafenib 81% (95%CI 69–96%) vs. placebo 36% (95%CI 22–57%) ORR of sorafenib 33% (95% CI 20–48%) vs. placebo 20% (95% CI 8–38%)
**ESTROGEN DRIVEN PATHWAY**					
*Anti-estrogen + NSAID* Tamoxifen + Sulindac	(89)	Phase 2	Pediatric DTF	59	*N* = 4 PR, *n* = 1 CR 2-year PFS 36%, OS 96%

*CI, confidence interval; CR, complete response; DTF, desmoid-type fibromatosis; GIST, gastro-intestinal stromal tumor; N, number of patients; NPRR, non-progressive response rate; NR, no response; ORR, objective response rate; ORS, overall response rate, OS, overall survival; PAR, progression arrest rate; PD, progressive disease; PFS, progression free survival; PR, partial response; SD, stable disease; TS, treatment stop; TTF, time to treatment failure; TTP, Time to progression; TTR, time to recurrence*.

While the above-mentioned treatments, targeting Wnt/β-catenin targets constitute attractive therapeutic possibilities, no prospective clinical trials using these treatment strategies in sporadic DTF have been designed. Experimental inhibitors of Wnt/β-catenin signaling have been developed, however, systemic abolition of Wnt secretion is not preferable since this will result in defects in gut homeostasis, affects the immune system and affects both ß-catenin-dependent and independent Wnt signaling [reviewed by Zimmerli et al. (90) and Enzo et al. (91)]. Figure 1 displays the Wnt/β-catenin signaling pathway and putative drug targets in the context of DTF.

## The Hedgehog Signaling Pathway in Desmoid-Type Fibromatosis

### The Hedgehog Signaling Pathway

The Hedgehog (Hh) signaling pathway plays an essential role in embryonic development, in adult tissue homeostasis, tissue renewal and tissue regeneration. Precursor proteins of Hh ligands, including Sonic (Shh), Indian (Ihh), and Desert (Dhh), undergo autocatalytic cleavage and cholesterol alterations at the carboxy terminal end, and palmitoylation at their amino terminal end. This process results in a dually-lipidated protein, which is released from the secreting cell surface. Subsequently, the Hh ligands interact with cell surface proteins like Glypican and the proteins of the heparin sulfate proteoglycan family enhancing their stability and promoting internalization when bound to Patched (PTCH1). Binding of Hh proteins to the canonical receptor PTCH1 and to co-receptors GAS1, BOC, and CDON initiates Hh signaling. This results in the release of PTCH1 mediated repression of the transmembrane protein Smoothened (SMO), a G-protein coupled receptor (GPCR)-like protein, which consequently leads to an accumulation of SMO in the cilia and phosphorylation of its cytoplasmic tail. Smoothened, regulates the downstream signal transduction which dissociates glioma associated oncogene (GLI) proteins, from kinesin-family protein, KIF7 and SUFU. GLI proteins serve as bifunctional transcription factors, capable of activating and repressing transcription, and form a key intracellular component of the Hh pathway [reviewed by Wu et al. (92) and Briscoe and Thérond (93)].

### The Hedgehog Signaling Pathway in Cancer

Aberrant Hh signaling in cancer is attributed to an increased endogenous Hh ligand expression, or to activating mutations of Hh pathway components [reviewed by Wu et al. (92)]. Aberrant uncontrolled activation of Hh has been described in numerous tumor types including; rhabdomyosarcoma (94), colorectal cancer (95), basal cell carcinoma (96), and medulloblastoma (97).

### The Hedgehog Signaling Pathway and Its Role and Therapeutic Potential in Desmoid-Type Fibromatosis

**Figure 2:**
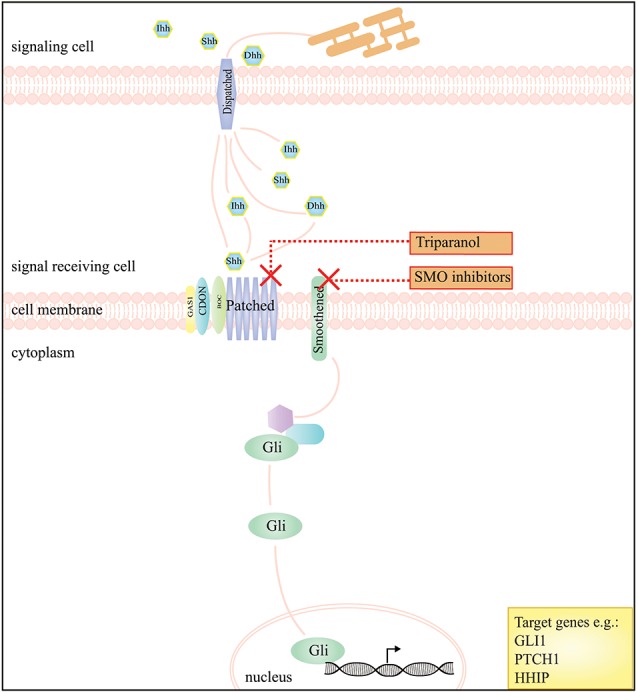
A schematic presentation of the Hedgehog signaling pathway and the drugs that interfere with this pathway in DTF. The graph depicts that inhibition of the Hedgehog pathway, by SMO inhibitors, works by blockage of Smoothened (SMO), a key regulator of downstream signaling by GLI transcription factors. The compound triparanol is known for inhibition of the cholesterol biosynthesis but can also interfere with Hedgehog signaling molecules including the Hedgehog ligand receptor Patched 1.

As the Hh pathway has the ability to maintain mesenchymal progenitor cells in a less differentiated state with greater proliferative capacity, it is possible that it influences proliferation of DTF cells in a similar manner because of the mesenchymal origin of these cells (61). Ghanbari et al. showed that Hh signaling is active in DTF by identifying a significant upregulation of Hh target genes *GLI1, PTCH1*, and *Hedgehog interacting protein* (*HHIP*) in human DTF samples compared to adjacent normal tissues. Additionally it was demonstrated that expression of *Gli1, Gli2*, and *Ptch1* in mouse (*Apc*^+/1638*N*^*)* tumors was upregulated compared to normal tissue. *In vivo*, pharmacological inhibition of Hh with triparanol, which works by interference with the post-translational modification of Hh signaling molecules and with the sterol-sensing domain of the receptor PTCH1, led to a reduction in tumor volume in *Apc*^+/1638*N*^ mice. Genetic approaches to reduce Hh signaling in DTF, using *Apc*^+/^^1638N^; *Gli2*^+/−^ mouse models, gave rise to the development of fewer and smaller tumors (Table 1) (61, 98). Currently, inhibition of the Hh signaling pathway acts via the pharmacological inhibition of SMO, however no clinical trials studying Hh inhibitors in DTF have been carried out. Figure 2 displays the Hh pathway and proposed working mechanism of target drugs in DTF.

## The Notch Signaling Pathway in Desmoid-Type Fibromatosis

### The Notch Signaling Pathway

Notch signaling is essential for regulating cell-fate during tissue development and for managing cell proliferation, differentiation and survival, neurogenesis and homeostasis in adult tissues [reviewed by Artavanis-Tsakonas et al. (99)]. There are four mammalian transmembrane Notch receptors (Notch receptor family type 1–4; NOTCH 1–4). Each receptor is a Ca^2+^-stabilized heterodimer containing three domains: an extracellular (NECD), a transmembrane (NTMD) and an intracellular domain (NICD) [reviewed by Takebe et al. (100)]. These receptors can interact with ligands; members of the Delta-like (DLL1, DLL3, and DLL4), and the Jagged (JAG1 and JAG2) families. In case of ligand binding, the receptor undergoes two processing steps. The first cleavage is mediated by a member of the disintegrin and metalloproteinase family (ADAM10 or ADAM17) and releases the NECD which remains bound to its ligand and is internalized by endocytosis in the cell that sends the signal. Subsequently in the receiving cell, a presenilin-dependent γ-secretase complex, removes the NICD from the NTMD. This NICD is translocated into the nucleus where it interacts with the CSL (CBF1/Suppressor of hairless/Lag-1) repressor complex; converting it into an activation complex that interacts with a co-activator protein mastermind-like 1 (MAML1). These interactions results in the transcriptional activation of several Notch target genes, such as *MYC, p21, HRT*, Notch receptors, Notch ligands, *cyclin D1*, and *HES*-family members [reviewed by Takebe et al. (100) and Ranganathan et al. (101)].

### The Notch Signaling Pathway in Cancer

Deregulation of the Notch signaling pathway is described in hematologic malignancies, notably T-cell acute lymphoblastic leukemia which harbors an activating mutation in NOTCH1 that result in a constitutive Notch signaling pathway activity (102). Although activating mutations in members of the Notch family are uncommon in solid tumors, Notch signaling may play a role in tumorigenesis [reviewed by Egloff and Grandis (103)]. For example, NOTCH3 transcript and protein levels are upregulated in a subset of colorectal cancers promoting tumor growth (104).

### The Notch Signaling Pathway and Its Role and Therapeutic Potential in Desmoid-Type Fibromatosis

Inhibition of Notch signaling forms an appealing therapeutic approach. Small molecular inhibitors, including γ-secretase inhibitors (GSI), siRNAs, and monoclonal antibodies against Notch receptors and ligands have been developed [reviewed by Yuan et al. (105)]. Particularly GSI's are of interest as these drugs inhibit the final Notch processing step by which NICD is released to act in the nucleus, consequently blocking Notch signaling. A number of GSI's (e.g., MK-0752 and RO4929097) have already been studied in solid cancers other than DTF in early phase clinical trials (106, 107).

**Figure 3:**
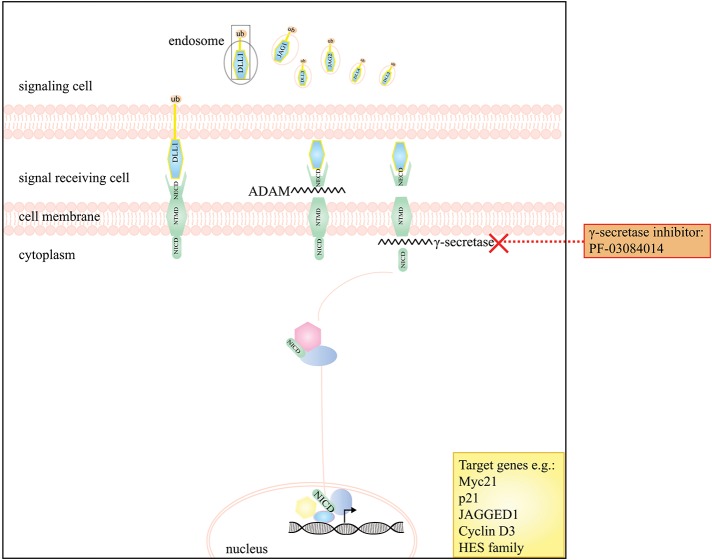
A schematic presentation of the Notch signaling pathway and the drugs that interfere with this pathway in DTF. The graph depicts that the Notch pathway can be targeted by the use of γ-secretase inhibitors e.g. PF-03084014.

Few studies investigated the role of the Notch signaling in DTF, however, DTF tumors have been shown to express NOTCH1 and its downstream target HES1 (108). Preliminary evidence, from a phase 1 clinical trial indicated a partial response in five out of seven DTF patients to the oral GSI PF-03084014 (Table 2) (109). This prompted an *in vitro* study performed by Shang et al. which demonstrated a significant higher expression of nuclear HES1 in DTF tissues compared to scar tissue by IHC and reported expression of NOTCH1, JAGGED1, and HES1 in DTF cells by Western Blot analysis. Additionally, it was demonstrated that PF-03084014, decreased NICD and HES1 expression in a dose dependent manner in DTF cells, and that Notch signaling inhibition contributed to impaired DTF cell proliferation by inducing a cell cycle G1 arrest and decreasing migration and invasion (Table 1) (62). Two other clinical trials (a phase 1 trial with seven DTF patients and phase II trial with 17 DTF patients) showed promising results with a significant part of patients experiencing partial response or stable disease (Table 2) (77, 78). Figure 3 displays the Notch signaling pathway and putative drug targets in the context of DTF.

## The JAK/STAT Signaling Pathway in Desmoid-Type Fibromatosis

### The JAK/STAT Signaling Pathway

The Janus-activated kinase (JAK) and signal transducer and activator of transcription (STAT) signaling pathway regulates cell proliferation, survival, differentiation, migration and apoptosis, and has a role in pathogen resistance. The JAK/STAT pathway is the main signaling mechanism for many cytokines and growth factors. A variety of ligands e.g., interferon-α/interferon-ß (IFN), secreted by leukocytes (IFN-α), fibroblasts (IFN-ß), and various other cells involved in immune responses, and interleukins (IL) together with their cognate receptors, stimulate the pathway. Ligand binding causes receptor dimerization and subsequent activation of the JAK tyrosine kinases associated with the cytoplasmic domains of the receptor. JAKs in close proximity are trans-phosphorylated and phosphorylate the receptors. The phosphorylated receptor sites can then serve as docking sites for cytoplasmic transcriptions factors (STATs). These STATs become phosphorylated by JAKs, dimerize and are translocated into the nucleus where they activate or repress the transcription of target genes [reviewed by O'Shea et al. (110)].

### The JAK/STAT Signaling Pathway in Cancer

Dysregulation of the JAK/STAT signaling pathway has been observed in several cancers including hematological and solid malignancies, such as breast [reviewed by Banerjee and Resat (111)] and prostate cancer [reviewed by Bishop et al. (112)]. Hallmarks of JAK/STAT dysregulation are: aberrant cytokine production, the occurrence of activating *JAK* mutations or mutations in other upstream oncogenes, and activating mutations in STAT [reviewed by O'Shea et al. (110)].

### The JAK/STAT Signaling Pathway and Its Role and Therapeutic Potential in Desmoid-Type Fibromatosis

In both human DTF samples and murine DTF models (*Apc/Apc*^1638*N*^), an increased expression of type 1 IFN response genes (e.g., *MxA, MxB, IFITI1*, and *IFNAR1*) have been identified suggesting an activated JAK/STAT signaling. Genes activated by this signaling pathway, have been shown to have an anti-proliferative effect on DTF cells and normal fibroblasts (Table 1) (63). Regression of DTF after treatment with IFN has been described in several case reports (113–116). A retrospective study by Leithner et al. examined 13 DTF patients receiving IFN-α ± tretinoin [a natural metabolite of vitamin A (retinol)]. This study indicated no evidence of disease in seven out of nine patients (adjuvant group), a mean disease-free interval of 22 months (±18 months), and progressive disease in two patients. Stabilization of DTF occurred in four patients which received IFN-α ± tretinoin (117). Despite these encouraging results, no prospective clinical trials in DTF have been carried out yet.

## The PI3 Kinase/Akt and mtor Signaling Pathways in Desmoid-Type Fibromatosis

### The PI3 Kinase/AKT and mTOR Signaling Pathways

The phosphoinositide 3 (PI3) kinase signaling pathway plays a critical role in various cellular processes like cell growth, survival, proliferation, metabolism and differentiation [reviewed by Engelmann et al. (118)]. The pathway is activated by plasma membrane proteins including receptor tyrosine kinases, integrins, B- and T-cell receptors, cytokine receptors and GPCRs and entails the formation of membrane-bound phosphatidylinositol-3,3,5-triphosphate (PIP3) by the enzyme PI3 kinase. Proteins that harbor a pleckstrin-homology (PH) domain like AKT (protein kinase B or PKB) and PDK1 bind to the 3-phosphoinositides on the membrane. Subsequent phosphorylation of AKT at the Thr308 and Ser473 residues, by PDK1 and mTORC2, respectively, fully activates its serine/threonine kinase potential. AKT consequently phosphorylates many downstream substrates, thereby regulating various cellular functions [reviewed by Hers et al. (119)]. Importantly, AKT also leads to downstream activation of the mTOR complex 1 (mTORC1) pathway by phosphorylation of its negative regulators TSC2 and PRAS40. Activation of this complex provides a growth advantage for cells, as mTORC1 is critical for cell maintenance by sensing nutritional and environmental cues and responding by inhibiting autophagy and regulating translation, thereby stimulating cell growth and proliferation [reviewed by Hers et al. (119) and Dowling et al. (120)].

### The PI3 Kinase/AKT and mTOR Signaling Pathways in Cancer

Dysregulation of the PI3 kinase/AKT signaling pathway is frequently encountered in cancers and facilitates tumorigenesis. Over-activation of AKT may be caused by the presence of gain-of-function mutations in PI3K subunits, or loss-of- function mutations in Phosphatase and Tensin homolog deleted from chromosome 10 (PTEN) or PTEN expression loss. PTEN, a tumor suppressor is a lipid phosphatase, negatively regulating AKT by dephosphorylation of PIP3. Alternatively, overexpression or activating mutations in tyrosine kinase receptors and their ligands, as well as the interaction of Ras with PI3K, can excite AKT activity [reviewed by Brugge et al. (121) and Keniry and Parsons (122)].

### The PI3 Kinase/AKT and mTOR Signaling Pathways and Their Role and Therapeutic Potential in Desmoid-Type Fibromatosis

The relationship between DTF and the PI3 kinase/AKT and the mTOR signaling pathways has not been extensively studied. Immunohistochemical analysis of 29 DTF tumor samples indicated the expression of ß-catenin and PDGFR-ß in all samples. No expression could be detected of PDGFR-α and phospho-Ser-473, suggesting inactive AKT signaling (123). Meazza et al. showed that a substantial part of pediatric DTF cases had an E17K mutation in either AKT1 (eight out of 28; 31%), however no AKT1 mutations were observed in adult DTF cases (*n* = 33) (124). Interestingly, DTF patients with an E17K AKT1 mutation had a longer recurrence free survival rate in agreement with the mutation-induced stimulation of downstream AKT signaling.

Recently, Rosenberg et al. reported the antitumor effect of sorafenib, a multi-kinase inhibitor that targets multiple tyrosine kinases (e.g., VEGFR, c-Kit, and PDGFR) expressed on patient derived DTF cell lines (Table 1). It was found that sorafenib decreased proliferation and invasion in a dose dependent manner and that the Ras/MEK/ERK and the PI3 kinase/AKT/mTOR signaling pathway were affected. Additionally, they investigated the efficacy of everolimus, an mTOR inhibitor, as monotherapy and in combination with sorafenib, and found no synergistic or additive inhibitory effect on cellular proliferation (Table 1) (64). Cates et al. compared the expression profiles of selected receptor- and non-receptor tyrosine kinases and downstream effectors of signaling activity in DTF (*n* = 27), reactive scars (*n* = 14) and fibrous tissue (*n* = 6) (Table 1). PDGFR-β, FAK 1 and MET were detected by IHC in almost all DTFs and scar tissues and in at least half (PDGFR-β, FAK1) or none (MET) of the fibrous control tissue. Of note, AKT was phosphorylated in 56% of DTF samples, but significantly higher levels were observed in scar tissues and only low levels were observed in a subset of fibrous tissues (125).

Inhibition of various tyrosine kinases including PDGFR with imatinib is a promising treatment strategy for DTF. Imatinib is effective in other solid tumors of mesenchymal origin like gastrointestinal stromal tumors (GISTs) and dermatofibrosarcoma protuberans, in which it targets KIT or PDGFR-α/PDGFR-ß [reviewed by Kosela-Paterczy and Rutkowski (126) and Casali et al. (127)]. The efficacy of imatinib (either alone or combined with other treatment) was observed in several early phase clinical studies with DTF patients (Table 2) (79–84). Chugh et al. and Penel et al. reported a 1-year progression free survival (PFS) of 66% and 67%, respectively. However, the results of these studies should be interpreted with caution, since the majority of patients included in these trials received other treatments prior to treatment with imatinib (Table 2) Additionally, the relevant targets of imatinib in DTF remain unclear (81, 123). Two phase II clinical trials, that included DTF patients, studied the effect of sunitinib (Table 2). George et al. did not detect a response to sunitinib in the single DTF patient that was included in their study (85). In contrast, a study by Jo et al. showed a 2-year PFS of 75% in 19 patients with advanced DTF (86). A phase I study, investigating the use of sorafenib in pediatric solid malignancies, of which two patients had DTF, showed a partial response on sorafenib in one patient (87). Recently published results from the phase 3 clinical trial (NCT02066181; sorafenib vs. placebo for advanced and refractory DTF) showed a 2-year PFS rate of 81% (95% confidence interval [CI] 69–96) with an objective response rate (ORR) of 33% (95% CI, 20–48) in the sorafenib group vs. 2-year PFS of 36% (95% CI, 22–57) and an ORR of 20% (95% CI, 8–38) in the placebo group (88). The biological mechanisms underlying the activity of sorafenib in DTF remain unclear because sorafenib targets multiple tyrosine kinases thereby affecting multiple pathways.

The use of imatinib, sunitinib and sorafenib, led to study the tyrosine kinase inhibitor (TKI) pazopanib in the setting of DTF. Pazopanib targets the VEGFR1-3, PDGFR-α, and PDGFR-ß, amongst others. A retrospective study by Szucs et al., described a partial response in three out of eight patients with DTF and stable disease in five out of eight patients with a median PFS of 13.5 months (128). Another retrospective study in adolescents and young adults with DTF by Agresta et al. reported tumor reduction after pazopanib use with only mild toxicities (Table 2) (129). One clinical trial is currently ongoing to investigate TKIs in the setting of DTF; pazopanib (NCT01876082, phase 2 study) (Table 3) (134).

**Table 3:** Overview of ongoing clinical trials with targeted drugs in DTF.

**Drug**	**References**	**NCT#**	**Setting**	**Current status**
**PI3 KINASE/AKT/mTOR SIGNALING PATHWAY**				
*Receptor Tyrosine Kinase inhibitor* Imatinib + vactosertib	(130)	NCT03802084	Phase 1/2 clinical trial	Not yet recruiting
*mTOR inhibitor* Sirolimus	(131)	NCT01265030	Phase 1 clinical trial Phase 2 clinical trial (children and young adults)	Recruiting
**NOTCH SIGNALING PATHWAY**				
*Notch inhibitor* PF-03084014	(132)	NCT01981551	Phase 2 clinical trial	Active, not recruiting
	(133)	NCT03785964	Phase 3 clinical trial	Not yet recruiting
**GROWTH REGULATORY SIGNALING PATHWAY**				
*PDGF-α/PDGF-ß/VEGFR* Pazopanib	(134)	NCT01876082	Phase 2 clinical trial	Recruiting

## The Growth Factor Regulatory Signaling Pathways in Desmoid-Type Fibromatosis

### The Growth Factor Regulatory Signaling Pathways

The superfamily of transforming growth factor-ß (TGF-ß) regulates cell proliferation, differentiation, apoptosis and development. Two ligand subfamilies are recognized; the TGF-β nodal subfamily, and bone morphogenetic protein (BMP) subfamily. Ligand binding of either TGF-β ligands or BMP ligands facilitates the oligomerization of type I and type II serine/threonine receptor kinases. In case of signaling, intracellular effectors R-SMAD's, are phosphorylated in the cytoplasm whereupon they partner with SMAD4 and translocate to the nucleus. In the nucleus they regulate, in conjunction with transcription factors/corepressors or co-activators, the transcription of TGF-ß target genes. Growth factor signaling pathways are initiated by various growth factors (e.g., insulin-like growth factors, platelet-derived growth factor, and hepatocyte growth factor) and induce phosphorylation of downstream targets via activation of their associated receptor tyrosine kinases. The signal is transduced through various intracellular intermediate molecules, frequently including PI3 kinase/AKT and Ras/Raf/MAPK signaling pathways to ultimately affect gene expression [reviewed by Massague (135)].

### The Growth Factor Regulatory Signaling Pathways and Their Role and Therapeutic Potential in Desmoid-Type Fibromatosis

The role of TGF-ß in DTF has been established by the expression of TGF-ß target genes (e.g. several collagen types and metalloproteinases) and by the upregulation of TGF-β signaling pathway components (phospho-SMAD2 and phospho-SMAD3, α-SMA and PAI1) in comparison to normal fascia (66). Mignemi et al. investigated TGF-ß signaling comparing DTF tissue with hypertrophic scars and fibrous tissue in human samples. It was discovered that the levels of TGF-ß receptor type 1 were similar in DTF and scar tissue, but that this receptor could not be detected in fibrous tissue. Phosphorylated SMAD2/3 could be detected in the majority of DTF samples (74%) but only in a minority of scar tissues (29%) and not at all in fibrous tissue (136). Additionally, TGF-ß stimulates β-catenin transcriptional activity, which indicates that this growth factor might play an important role in the development of DTF (66). Multiple studies reported overexpression of platelet derived growth factors and their associated receptors (PDGFα, PDGFRα, PDGFß, and PDGFRß) in neoplastic fibrous proliferations including DTF and myofibromatoses (137, 138).

Various studies investigated the effect of TFG-ß and epidermal growth factor (EGF) on DTF cell lines and stimulation with these cytokines caused up- and down regulation of various target genes (e.g., *SMAD4*), changes in β-catenin levels, and increased production of glycosaminoglycan and collagen. Additionally, treatment with EGF increased DTF cell motility (Table 1) (65–68).

Cross-talk between the insulin-like growth factor (IGF) and the estrogen receptor (ER) mediated signaling has been demonstrated in breast-cancer cells. Activation of MAPK, which is located downstream of IGF-1, enhances ER induced transcription via ER phosphorylation. Therefore, it is presumed that growth factor signaling pathways and the estrogen pathways complement and overlap each other [reviewed by Dhingra (139)]. Toremifene, a drug which also inhibits collagen synthesis and protein kinase C, works on both the growth factor regulatory signaling pathway and the estrogen pathway and will be discussed in the next section.

## The Role of the Estrogen Driven Pathway in Desmoid-Type Fibromatosis

### The Estrogen Driven Pathway

Estrogens affect various physiological processes that concern the development of the reproductive system and several reproductive functions. The role of estrogens in cancer has been described in breast cancer and uterine tumors [reviewed by Dhingra (139)]. Two subtypes of estrogen receptors (ER) haven been identified; ER-α (ESR1) and ER-ß (ESR2) to which estrogens can bind. Without the ligand, the intracellular ER is considered to be in an inactive state, forming a complex with two heat shock proteins (Hsp90 and Hsp56) and various other proteins. Upon binding of the ligand estrogen, Hsp90 is detached from the complex which leads to ER phosphorylation. Next, the ER dimerizes and translocates to the nucleus where it can interact with specific DNA sequences, estrogen response elements (ERE), causing transcriptional activation [reviewed by Dhingra (139) and Picariello et al. (140)].

### The Estrogen Driven Pathway and Its Role and Therapeutic Potential in Desmoid-Type Fibromatosis

The role of sex hormones, particularly estrogen, in DTF tumorigenesis is primarily based on clinical observations, and prompted the use of anti-hormonal agents. DTF arises frequently in females at the reproductive age (4). Moreover, tumor growth during pregnancy, accelerated tumor growth by using oral contraceptives and tumor regression in menopause are reported (10, 141). Although DTF does not express ER-α, Deyrup et al. discovered that DTF tumors express ER-ß (142). The involvement of estrogens in DTF offers a variety of interesting hormone related drugs which can be a potential treatment for DTF. Tamoxifen is most extensively studied in the setting of DTF and is often used in various dosages in combination with other drugs, frequently NSAIDs, like sulindac [reviewed by Bocale et al. (143)]. A meta-analysis by Bocale and Rotelli et al. verified that tamoxifen, administered as a single agent, gave an overall response rate (partial or complete response) of 58% (22 out of 38 patients). In combination with NSAIDs, this response rate decreased to 35% (143). Likewise, toremifene has been shown to have an antitumor effect in DTF (71, 144). Although it does not influence cell proliferation, toremifene decreased the total amount of glycosaminoglycans (GAGs), TGFß1, collagen and fibronectin levels and it diminished the affinity of type I and II TGFβ1 receptors for ^125^I-TGF-ß1 (Table 1) (65, 70). Toremifene in retrospective clinical studies, administered alone or in combination with other drugs like melatonin, sulindac or IFN-α, yielded an overall complete and partial response rate of 56%. When comparing tamoxifen and toremifene, used as a single agent, no differences in overall response rate were found [reviewed by Bocale et al. (143)]. Raloxifene, a drug initially developed for treating chronic osteoporosis, was administered to 13 patients with FAP-related DTF which were refractory to other treatments. Eight patients displayed a complete remission and a partial response was seen in five cases (145). Despite a clear involvement of estrogens in DTF, response rates to anti-estrogen agents vary and the number of prospective clinical studies is still limited. One phase II trial combined sulindac (NSAID) with tamoxifen in pediatric DTF patients and showed a 1-year PFS of 36% (Table 2) (89).

## Future Directions and Conclusion

This review aims to provide a summary of the current knowledge of important, cancer-related signaling pathways in the setting of DTF. The role of Wnt/β-catenin signaling in DTF has been firmly established in numerous studies, showing the presence of β-catenin signaling enhancing mutations in the vast majority of tumors. Therapeutic options targeting the Wnt/β-catenin signaling pathway remain scarce and are not yet widely tested in the clinical setting for DTF. Several clinical trials, targeting other signaling pathways, like Notch and Hedgehog, are currently ongoing, but few study the contribution of these pathways to DTF tumorigenesis.

A major challenge remains to study DTF in the preclinical setting. This is partly due to the rarity of the tumor type, but also to the limited availability of DTF cell lines and other cell and animal models (3). Culturing a fresh DTF resection specimen, inevitably leads to an overgrowth of WT fibroblasts and concomitant loss of DTF tumor cells. Separating tumor cells from their surrounding stromal cells remains challenging and time consuming. Even if a “pure” DTF cell line is obtained, DTF tumor cells often reach senescence after several passages. To our knowledge, no studies investigating the effect of immortalization protocols on primary DTF cells have been published yet. Experimenting with primary cultures, consisting of both stromal cells and tumor cells, is an alternative but has its drawbacks. Additionally, representative cellular and animal models of DTF (e.g., organoids or mice expressing mutated *CTNNB1*) in relevant tissues are difficult to generate and expensive to maintain. The existing *Apc*^+^*/Apc*^1638*N*^ mouse model has already been proven as a useful model for FAP-associated DTF and is often used as a tumor model for non-FAP related DTF (Table 1) (61, 146). A mouse DTF model based on specific β-catenin mutations is, to our knowledge, currently not available. A recently developed genetically engineered *Xenopus tropicalis* model harboring a mutated *APC*, may yield another DTF tumor model that can be exploited as a platform to define novel therapeutic targets and preclinical validation studies (147). Well-defined preclinical models are as necessary as well-annotated large series of DTF tumor samples, to better understand DTF biology and to provide experimental support and rationale for translational research investigating the inhibition of signaling pathways in DTF.

Additionally, signaling pathways are often seen as separate entities, however, in reality cross-talk occurs between different pathways. The precise interactions between different signaling- and biochemical pathways is complex and still poorly understood. Aberrant signaling of one pathway can often be corrected via compensatory mechanisms in another pathway (148). In DTF tumors and cell lines, the Wnt, Notch and Hh signaling pathways have been shown to be involved in cross-talk, implicating optimal therapeutic efficacy, is reached when all interacting pathways are inhibited in a combinatorial approach (149). Future studies should not focus on individual signaling cascades but rather on the simultaneous inhibition of multiple pathways.

Furthermore, clinical studies to evaluate the efficacy of systemic and targeted treatments without any randomization procedures remain challenging in current clinical practice as they are often difficult to interpret. Due to the unpredictable growth behavior of DTF with reports of spontaneous regression without treatment and stable disease, it is difficult to distinguish the true treatment effects from natural growth behavior. Moreover, the design of randomized controlled trials might be restricted by the rarity of the disease and the small number of DTF patients with an indication for systemic treatment. Despite these challenges, future studies should include signaling pathways other than Wnt/β-catenin signaling to uncover additional driver genes and pathways in DTF and to clarify the potential working mechanisms of target drugs in the setting of DTF.

## Ethics Statement

This article does not contain any studies with human participants or animals performed by any of the authors. For this type of study formal consent is not required.

## Author Contributions

MT, DG, CV, SS, and EW conceptualized and designed the study. MT wrote the first draft of the manuscript. MT, RS, DG, CV, SS, and EW wrote sections of the manuscript. All authors contributed to the manuscript revision, read and approved the submitted version.

### Conflict of Interest Statement

The authors declare that the research was conducted in the absence of any commercial or financial relationships that could be construed as a potential conflict of interest.
