# Supplementary material for: Activated Signaling Pathways and Targeted Therapies in Desmoid-Type Fibromatosis: A Literature Review
Source: Front Oncol. 2019 May 17;9:397. doi: 10.3389/fonc.2019.00397 (PMC6534064; doi:10.3389/fonc.2019.00397)
Supplement: Supplementary file 1 [file Table_1.DOCX]

**Appendix 1**

**List of abbreviations used in this manuscript**

| **ADAM** | A disintegrin and metalloproteinase |
| --- | --- |
| **AKT** | AKT8 virus oncogene cellular homolog |
| **APC** | Adenomatous polyposis coli |
| **BOC** | Brother of CDON |
| **BMP** | Bone morphogenetic protein |
| **CDON** | Cell adhesion associated, oncogene regulated |
| **CK** | Casein kinase |
| **COX** | Cyclooxygenase |
| **CSL** | CBF1/ Suppressor of hairless/Lag-1 |
| **CR** | Complete response |
| **CRC** | Colorectal cancer |
| **DFMO** | Difluoromethylornithine |
| **DFU** | Selective COX-2 blocker (5,5-dimethyl-3-(3-uorophenyl)4-(4-methylsulphonyl)phenyl-2(5H)-furon one) |
| **Dhh** | Desert hedgehog |
| **DLL** | Delta-Like Canonical Notch Ligand |
| **DVL** | Dishevelled protein |
| **DTF** | Desmoid-type fibromatosis |
| **EGF** | Epidermal growth facto |
| **ER** | Estrogen receptors |
| **ERE** | Estrogen response elements |
| **ERK** | extracellular signal-regulated kinase |
| **FAP** | Familial adenomatous polyposis |
| **GAG** | Glycosaminoglycans |
| **GAS** | Growth arrest specific |
| **GIST** | Gastro-intestinal stromal tumors |
| **GLI** | Glioma-associated oncogene |
| **GPCR** | G-protein coupled receptor |
| **GSI** | Gamma-Secretase Inhibitors |
| **GSK** | Glycogen synthase kinase |
| **HES** | Hairy enhancer of split /HES Family BHLH Transcription Factor |
| **Hh** | Hedgehog |
| **HHIP** | Hedgehog interacting protein |
| **Hsp** | Heat shock proteins |
| **IGF** | Insulin growth factor |
| **IFN** | Interferon |
| **Ihh** | Indian hedgehog |
| **IL** | Interleukins |
| **Jag** | Jagged |
| **JAK** | Janus-activated kinase |
| **KIF7** | Kinesin family protein |
| **KIT** | v-KIT Hardy-Zuckerman 4 Feline Sarcoma viral oncogene homolog/ KIT oncogene |
| **LEF** | Lymphoid enhancer factor |
| **LRP** | Lipoprotein receptor-related protein |
| **MAML1** | Mastermind-like 1 |
| **MAPK** | Mitogen-activated protein kinase |
| **MEK** | MAPK/Erk kinase |
| **MET** | Hepatocyte growth factor receptor |
| **MMP** | Matrix Metalloproteinase |
| **mTOR** | Mechanistic Target Of Rapamycin |
| **NECD** | Notch extra-cellular domain |
| **NF** | Normal fibroblast |
| **NICD** | Notch intra-cellular domain |
| **NPRR** | Non-progressive response rate |
| **NR** | No response |
| **NTMD** | Notch transmembrane domain |
| **NSAID** | Non-steroidal anti-inflammatory drug |
| **ORR** | Objective response rate |
| **OS** | Overall survival |
| **PAR** | Progression arrest rate |
| **PCC** | Primary cell culture |
| **PD** | Progressive disease |
| **PDK** | Phosphoinositide-dependent kinase |
| **PDGF(R) ɑ/ß** | Platelet Derived Growth Factor (Receptor) ɑ/ß |
| **PFS** | Progression free survival |
| **PH** | Pleckstrin-homolog |
| **PI3** | Phosphatidylinositol 3 |
| **PI3K** | Phosphatidylinositol 3-kinase |
| **PIP3** | Phosphatidylinositol (3,4,5)-triphosphate |
| **PR** | Partial response |
| **PTCH1** | Patched 1 |
| **PTEN** | Phosphatase and tensin homolog deleted on chromosome ten (10) |
| **RTK** | Receptor tyrosine kinase |
| **SD** | Stable disease |
| **Shh** | Sonic hedgehog |
| **SMAD** | SMA- and MAD-related protein / SMAD Family member |
| **SMO** | Smoothened |
| **STAT** | Signal Transducer and Activator of Transcription |
| **SUFU** | Suppressor of fused homolog |
| **TCF** | T-cell factor |
| **TGF-ß** | Transforming growth factor-ß |
| **TKI** | Tyrosin kinase inhibitor |
| **TS** | Treatment stop |
| **TTF** | Time to treatment failure |
| **TTP** | Time to progress |
| **VEGF** | Vascular Endothelial Growth Factor |
| **WT** | Wild-type |
| **WISP** | Wnt-1 Inducible Signaling pathway Protein 1 |
| **Wnt** | Wingless |

|  |  |
| --- | --- |
